# Reviewer acknowledgement

**DOI:** 10.1186/s12974-016-0518-6

**Published:** 2016-03-08

**Authors:** Sue T Griffin, Robert E Mrak

**Affiliations:** Department of Geriatrics, University of Arkansas for Medical Sciences, Little Rock, Arkansas USA; Department of Pathology, University of Toledo Health Sciences Campus, Toledo, USA

## Abstract

The editors of *Journal of Neuroinflammation* would like to thank all our reviewers who have contributed to the journal in Volume 12 (2015).

**Lailla Abdullah**

United States of America

**Rosella Abeti**

United Kingdom

**Yousef Abu-Amer**

United States of America

**Dong Ahn**

Republic of Korea

**Saba Aïd**

France

**Yousef Al-Abed**

United States of America

**Philipp Albrecht**

Germany

**Jessy Alexander**

United States of America

**Lena Al-Harthi**

United States of America

**Artino Allen**

United States of America

**Joshua Allen**

United States of America

**Lydia Alvarez**

Spain

**Diana Amantea**

Italy

**Fumimasa Amaya**

Japan

**Winfried Amoaku**

United Kingdom

**Aileen Anderson**

Lebanon

**Anuska Andjelkovic Zochowska**

United States of America

**Zane Andrews**

Australia

**Juan Anguita**

Spain

**Daniel Anthony**

United Kingdom

**Ichio Aoki**

Japan

**Revital Aricha**

Israel

**Kiyoshi Ariizumi**

United States of America

**Regina Armstrong**

United States of America

**Rand Askalan**

Canada

**Stéphane Auvin**

France

**Hafidi Aziz**

France

**Ana Baamonde**

Spain

**Adam Bachstetter**

United States of America

**Ryan Bachtell**

United States of America

**Veerle Baekelandt**

Belgium

**Fulvio Baggi**

Italy

**David Baker**

United Kingdom

**Silvia Balosso**

Italy

**Giorgos Bamias**

Greece

**Banani Banerjee**

United States of America

**William Banks**

United States of America

**Steven Barger**

United States of America

**Anne Baron-Van Evercooren**

France

**Ruth Barrientos**

United States of America

**Anirban Basu**

India

**Jan Bauer**

Austria

**Ingo Bechmann**

Germany

**Andreas Beineke**

Germany

**Jean-Pierre Bellier**

Japan

**Kendra Bence**

United States of America

**Jeffrey Bennett**

United States of America

**Thomas Berger**

Austria

**Cornelia Bergmann**

United States of America

**Kristen Bernard**

United States of America

**Sonia Berrih-Aknin**

France

**Sonja Best**

United States of America

**Knut Biber**

Germany

**Roger Bick**

United States of America

**Paula Bickford**

United States of America

**Helle Bielefeldt-Ohmann**

Australia

**Stacy Bilbo**

United States of America

**Silvia Bisti**

Italy

**Gregory Bix**

United States of America

**Gregory Bix**

United States of America

**Klas Blomgren**

Sweden

**Mathew Blurton-Jones**

United States of America

**Juan Bolanos**

Spain

**Kathryn Bollinger**

United States of America

**Jessica Bolton**

United States of America

**Bruno Bonaz**

France

**Karin Borges**

Australia

**Cesar Borlongan**

United States of America

**Kenneth Bost**

United States of America

**Charles Bradberry**

United States of America

**Monika Bradl**

Austria

**Lars-Ove Brandenburg**

Germany

**David Braun**

United States of America

**Amy Brewster**

United States of America

**Kris Brickman**

United States of America

**Celia Brosnan**

United States of America

**Brad Broughton**

Australia

**Jonathan Brouillette**

Canada

**Guy Brown**

United Kingdom

**Joan Brown**

United States of America

**Wolfgang Brück**

Germany

**Luigi Bubacco**

Italy

**Marion Buckwalter**

United States of America

**Bruce Bunnell**

United States of America

**Irina Burd**

United States of America

**Ta Butterick**

United States of America

**Kimberly Byrnes**

United States of America

**Huaibin Cai**

United States of America

**Lilian Calderon-Garciduenas**

United States of America

**Patrick Calders**

Belgium

**Ruth Caldwell**

United States of America

**Sean Callanan**

Ireland

**Arezoo Campbell**

United States of America

**Matthew Campbell**

Ireland

**Eduardo Candelario-Jalil**

United States of America

**Ling Cao**

United States of America

**Jeffrey Capadona**

United States of America

**Daniel Carr**

United States of America

**Richard Carr**

Germany

**Monica Carson**

United States of America

**Anna Carta**

Italy

**Ignazio Castagliuolo**

Italy

**Bogdan Catalin**

Germany

**Paola Cavalcante**

Italy

**Paramita Chakrabarty**

United States of America

**Saikat Chakraborty**

United States of America

**Angel Chamorro**

Spain

**Andrew Chan**

Germany

**Alexandre Chan**

Singapore

**Gusharan Chana**

Australia

**Christiane Charriaut-Marlangue**

France

**Abhijit Chaudhuri**

United Kingdom

**Maxim Cheeran**

United States of America

**Sylvain Chemtob**

Canada

**Chu Chen**

United States of America

**Szu-Fu Chen**

Taiwan Republic of China

**Chun-Jung Chen**

Taiwan Republic of China

**Zhihong Chen**

United States of America

**Jonathan Cherry**

United States of America

**Glyn Chidlow**

Australia

**Isaac Chiu**

United States of America

**Sunghee Cho**

United States of America

**Sang-Ho Choi**

United States of America

**Michael Chopp**

United States of America

**Kon Chu**

Republic of Korea

**J. Clark**

United States of America

**Diego Clemente**

Spain

**Manola Comar**

Italy

**Colin Combs**

United States of America

**Phillippe Corcia**

France

**Peter Crack**

Australia

**David Cribbs**

United States of America

**Anne Cross**

United States of America

**E Cudaback**

United States of America

**Colm Cunningham**

Ireland

**Riccardo Cuppini**

Italy

**Daniela Curti**

Italy

**Krzysztof Czaja**

United States of America

**Endre Czeiter**

Hungary

**Cleide Da Silva**

United States of America

**Haibin Dai**

China

**Russell Dale**

Australia

**Robert Dantzer**

United States of America

**Kunjan Dave**

United States of America

**Terry Davidson**

United States of America

**Randall Davis**

United States of America

**Alejandro De Denicola**

Argentina

**Pierre De Graan**

Netherlands

**Joris De Man**

Belgium

**Rocío M. De Pablos**

Spain

**Tara De Silva**

United States of America

**Terrence Deak**

United States of America

**Rashid Deane**

United States of America

**Gregory Dekaban**

Canada

**Sharon Demorrow**

United States of America

**Gerald V. Denis**

United States of America

**Candan Depboylu**

Germany

**Matthew Derrick**

United States of America

**Ronald Deumens**

Belgium

**Kumlesh Dev**

Ireland

**Carlie Devries**

Netherlands

**Courtney Devries**

United States of America

**Cameron Dezfulian**

United States of America

**Barbara Di Benedetto**

Germany

**Betty Diamond**

United States of America

**Exuperio Diez-Tejedor**

Spain

**Christine D. Dijkstra**

Netherlands

**Charles Dinarello**

United States of America

**Kelley Dineley**

United States of America

**Yuchuan Ding**

United States of America

**Rosario Donato**

Italy

**Catherine Dostert**

Luxembourg

**Patrick Dougherty**

United States of America

**Sarah Doyle**

Ireland

**Alex Dregan**

United Kingdom

**Paul Drew**

United States of America

**Kirk Druey**

United States of America

**Ronald Duman**

United States of America

**Aaron Dumont**

United States of America

**Alexander Easton**

Canada

**David Edwards**

United States of America

**Arik Eisenkraft**

Israel

**Joseph El Khoury**

United States of America

**Najira El-Hage**

United States of America

**Ll Elias**

Brazil

**Michal Elovitz**

United States of America

**Azza El-Remessy**

United States of America

**Mitsuhiro Enomoto**

Japan

**Adviye Ergul**

United States of America

**Michelle Erickson**

United States of America

**Ana Esquifino**

Spain

**Elsa Fabbretti**

Slovenia

**Sarah Falk**

Denmark

**Brian Fallon**

United States of America

**Chen-Ming Fan**

United States of America

**Cinthia Farina**

Italy

**Krysten Farjo**

United States of America

**Geraldine Favrais**

France

**Douglas Feinstein**

United States of America

**Robert Felder**

United States of America

**Vicente Felipo**

Spain

**Hua Feng**

China

**Patricia Fernandez**

Panama

**Javier Fernandez-Ruiz**

Spain

**Elisabeth Fernell**

Sweden

**Donna Ferriero**

United States of America

**Bernd Fiebich**

Germany

**Stefano Fiorucci**

Italy

**Mark Fisher**

United States of America

**Loretta Flanagan-Cato**

United States of America

**Mary Ann Fletcher**

United States of America

**Willem-Jan Fokkink**

Netherlands

**Laura Fonken**

United States of America

**G Forloni**

Italy

**Carola Forster**

Germany

**Thomas C. Foster**

United States of America

**Fornai Francesco**

Italy

**Joseph Francis**

United States of America

**Martin Frasch**

Canada

**Sally Frautschy**

United States of America

**Gregory Freund**

United States of America

**Manuel Friese**

Germany

**John Fryer**

United States of America

**Wen-Mei Fu**

Taiwan Republic of China

**Dietmar Fuchs**

Austria

**Ken-Ichiro Fukuchi**

United States of America

**Roberto Furlan**

Italy

**Prasad Gabbita**

United States of America

**Bhakta Prasad Gaire**

Republic of Korea

**Rob Galinsky**

New Zealand

**Lucio Gama**

United States of America

**Me Gambuzza**

Italy

**Olgo Garaschuck**

Germany

**Lidia Garcia-Bonilla**

United States of America

**Borja García-Bueno**

Spain

**D Garcia-Ovejero**

Spain

**Gwenn Garden**

United States of America

**Peter Garred**

Denmark

**Peter Gaskill**

United States of America

**Julie Gavard**

France

**Harris Gelbard**

United States of America

**John Gensel**

United States of America

**Steve M Gentleman**

United Kingdom

**Igal Gery**

United States of America

**Simantini Ghosh**

United States of America

**Othman Ghribi**

United States of America

**Francesca Gilli**

United States of America

**Pietro Giusti**

Italy

**Jonathan Godbout**

United States of America

**Norbert Goebels**

Germany

**Beryl Gok**

United States of America

**Ralf Gold**

Germany

**Yona Goldshmit**

Israel

**Diego Gomez-Nicola**

United Kingdom

**Michael Goodman**

United States of America

**Nicolai Gorbunov**

United States of America

**Carmen Gottfried**

Brazil

**Peter Grace**

United States of America

**Peter Grace**

United States of America

**Manuel Graeber**

Australia

**Elizabeth Gray**

United Kingdom

**Kim Green**

United States of America

**Paul Green**

United States of America

**Kim Green**

United States of America

**Elisa Greggio**

Italy

**Diane Griffin**

United States of America

**Christian Grimm**

Switzerland

**Wolfgang Grisold**

Austria

**Floris Groenendaal**

Netherlands

**Antje Grosche**

Germany

**Ross Gruber**

United States of America

**Alexandra Grubman**

Australia

**Franz Grus**

Germany

**Jean-Charles Guéry**

France

**Alistair Gunn**

New Zealand

**Rainer Haberberger**

Australia

**Abigail Hackam**

United States of America

**Marc Halterman**

United States of America

**Khalid Hanafy**

United States of America

**Andrew Harkin**

Ireland

**Ashley Harms**

United States of America

**Marni Harris-White**

United States of America

**Higashida Haruhiro**

Japan

**David Hasan**

United States of America

**Tomoki Hashimoto**

United States of America

**Johnny He**

United States of America

**Shuhan He**

United States of America

**Johnny He**

United States of America

**Raimund Helbok**

Austria

**Michael Heneka**

Germany

**Michael Hennessy**

United States of America

**Kenneth Hensley**

United States of America

**Marina Hernandes**

United States of America

**Paco Herson**

United States of America

**David Hess**

United States of America

**James Hewett**

United States of America

**Sandra Hewett**

United States of America

**Hubertus Himmerich**

Germany

**Rabea Hinkel**

Germany

**Holly Hinson**

United States of America

**Yoshitaka Hirooka**

Japan

**Silke Hirsch**

Germany

**Roger Ho**

Singapore

**Shelley Hooks**

United States of America

**Douglas Hooper**

United States of America

**Kathryn Hopperton**

Canada

**Motohiro Horiuchi**

Japan

**Charles Howe**

United States of America

**Christine Hsieh**

United States of America

**Huijuan Hu**

United States of America

**Xiaoming Hu**

United States of America

**Rui Huang**

China

**Christian Humpel**

Austria

**Fumito Ichinose**

United States of America

**O Igwe**

United States of America

**Hiroko Ikeshima-Kataoka**

Japan

**Jeffrey Iliff**

United States of America

**Atsuko Inoue**

Japan

**David Irani**

United States of America

**Takayuki Itoh**

United States of America

**Jason Ivanusic**

Australia

**Koichi Iwata**

Japan

**Joel Jackobsson**

Sweden

**Sanjay Jain**

United States of America

**Tatjana Jakobs**

United States of America

**Krzysztof Janeczko**

Poland

**Glen Jeffery**

United Kingdom

**Hui-Rong Jiang**

United Kingdom

**Meiyan Jiang**

United States of America

**Erik Johnson**

United States of America

**John Johnson**

United States of America

**Aaron Johnson**

United States of America

**Nicole Jones**

Australia

**Ilona Joniec-Maciejak**

Poland

**Chung Ju**

Republic of Korea

**Robert Kahoud**

United States of America

**Ryuji Kaji**

Japan

**Shuji Kaneko**

Japan

**Scott Kanoski**

United States of America

**A. Kanthasamy**

United States of America

**William Karpus**

United States of America

**Toshihiko Katafuchi**

Japan

**Yasufumi Kataoka**

United States of America

**Hiroshi Katsuki**

Japan

**Anu Kauppinen**

Finland

**Syed Kazim**

United States of America

**Richard Keep**

United States of America

**Samia Khoury**

Lebanon

**Mahmoud Kiaei**

United States of America

**Anton Kichev**

United Kingdom

**Ho Jin Kim**

Republic of Korea

**Jung-Ae Kim**

Republic of Korea

**Hyoung-Chun Kim**

Republic of Korea

**Myeung Ju Kim**

Republic of Korea

**Nicholas King**

Australia

**T Kiyota**

United States of America

**Christoph Kleinschnitz**

Germany

**Ingo Kleiter**

Germany

**Firas Kobeissy**

United States of America

**Jae Young Koh**

Republic of Korea

**Hideo Kohno**

Japan

**Ron Kooijman**

Belgium

**Juerfen Kopitz**

Germany

**Thomas Korn**

Germany

**S. Korte**

Netherlands

**Elöd Körtvely**

Germany

**Robert Kraft**

Germany

**Chia-Yi Kuan**

United States of America

**Markus Kuehn**

United States of America

**Stefanie Kuerten**

Germany

**Patrick Kuery**

Germany

**Donald Kuhn**

United States of America

**Stephanie Kullmann**

Germany

**Anil Kumar**

United States of America

**Jinn-Rung Kuo**

Taiwan Republic of China

**Jacek Kurzepa**

Poland

**Katarzyna Kwiatkowska**

Poland

**Anne La Flamme**

New Zealand

**Jose L Labandeira-Garcia**

Spain

**Steve Lacroix**

Canada

**Mary Jo. Ladu**

United States of America

**Philip Landfield**

United States of America

**Gary Landreth**

United States of America

**Thomas Lane**

United States of America

**Thomas Langmann**

Germany

**Mj Lavoie**

United States of America

**Catherine Lawrence**

United Kingdom

**David Lawrence**

United States of America

**Marcus Lawson**

United States of America

**Eric Lazartegues**

United States of America

**Eric Lazartigues**

United States of America

**John Lee**

Australia

**Sung Joong Lee**

Republic of Korea

**Sung Joong Lee**

Republic of Korea

**Sunhee Lee**

United States of America

**Sunhee Lee**

United States of America

**Christian Lefebvre D'hellencourt**

France

**Donald Lehmann**

United Kingdom

**Helmar Lehmann**

Germany

**Michael Lehmann**

United States of America

**Emily Leibovitch**

United States of America

**Guilhian Leipnitz**

Brazil

**Marcel Leist**

Germany

**Vincent Lelievre**

France

**Lisa Leon**

United States of America

**Christina Leslie**

United States of America

**Michael Levy**

United States of America

**Patrick Lewis**

United Kingdom

**Frank Leypoldt**

Germany

**Didier Leys**

France

**Cai Li**

United States of America

**J Li**

United States of America

**Junfa Li**

China

**Jian Liang**

United States of America

**Daniel Liebl**

United States of America

**Fabian Liechti**

Switzerland

**Jihyeon Lim**

United States of America

**Charles L. Limoli**

United States of America

**Raija Lindberg**

Switzerland

**Dan Lindholm**

Finland

**Eng-Ang Ling**

Singapore

**Ralf Linker**

Germany

**Gregory Liou**

United States of America

**Rob Lisak**

United States of America

**Lucia Lisi**

Italy

**Fudong Liu**

United States of America

**Lixin Liu**

Canada

**Nk Liu**

United States of America

**Jialing Liu**

United States of America

**Yawei Liu**

Denmark

**Yutong Liu**

United States of America

**Jordi Llop**

Spain

**Sarah Elisabeth Lloyd**

United Kingdom

**Eng Lo**

United States of America

**David Loane**

United States of America

**David Loeffler**

United States of America

**James Lokensgard**

United States of America

**Ruben Lopez-Vales**

Spain

**Lisa Loram**

United States of America

**Seth Love**

United Kingdom

**Qingxian Lu**

United States of America

**Ru-Band Lu**

Taiwan Republic of China

**Paul Lucassen**

Netherlands

**Ralph Lucus**

Germany

**Jan Luenemann**

Switzerland

**Jia Luo**

United States of America

**Sarah Lutz**

United States of America

**Marina Lynch**

Ireland

**Daqing Ma**

United Kingdom

**Qingyi Ma**

China

**Halina Machelska**

Germany

**Andrew Maclean**

United States of America

**Akiko Maeda**

United States of America

**Sanjay Maggirwar**

United States of America

**Kathleen Maguire-Zeiss**

United States of America

**Pamela Maher**

United States of America

**Sabatino Maione**

Italy

**Marzia Malcangio**

United Kingdom

**Anne-Marie Malfait**

United States of America

**Carina Mallard**

Sweden

**Frederic Manfredsson**

United States of America

**Santhakumar Manicassamy**

United States of America

**Mark Mapstone**

United States of America

**Yannick Marchalant**

United States of America

**Nicola Marchi**

United States of America

**Carmela Marino**

Italy

**Daniel Marks**

United States of America

**Paola Marmiroli**

Italy

**Ian Marriott**

United States of America

**Thomas Martin**

United States of America

**J Martinez-Gonzalez**

Spain

**Ricardo Martinez-Zamudio**

France

**Rudolf Martini**

Germany

**Ángeles Martín-Requero**

Spain

**Eliezer Masliah**

United States of America

**Iara Massias-Reason**

Brazil

**Clayton Mathews**

United States of America

**Kaspar Matiasek**

Germany

**F Matsuda**

Japan

**Carlow Matute**

Spain

**Anne Kathrin Mausberg**

Germany

**Christian Mawrin**

Germany

**Audrey Mazarati**

United States of America

**Mervyn Maze**

United States of America

**Simon Mcarthur**

United Kingdom

**Louise Mccullough**

United States of America

**Robert Mccusker**

United States of America

**Braden Mcfarland**

United States of America

**James Mcginnis**

United States of America

**Declan Mckernan**

Ireland

**Gordon Meares**

United States of America

**Stephane Melik Parsadaniantz**

France

**Richard Mellanby**

United Kingdom

**Til Menge**

Germany

**Doron Merkler**

Germany

**Axel Methner**

Germany

**Jeffrey Meyer**

Canada

**Gerd Meyer Zu Horste**

Germany

**Geert Meyfroidt**

Belgium

**Jens Mikkelsen**

Denmark

**Djordje Miljkovic**

Serbia And Montenegro

**C Miller**

United States of America

**Donald Miller**

Canada

**Dennis Miller**

United States of America

**Robert Miller**

United States of America

**Richard Miller**

United States of America

**Rachel Miller**

United States of America

**Richard Milner**

United States of America

**Luisa Minghetti**

Italy

**Yasushi Miura**

Japan

**Takuro Miyazaki**

Japan

**Ehsan Moazen Zadeh**

Islamic Republic Of Iran

**Alon Monsonego**

Israel

**Joan Montaner**

Spain

**Darren Moore**

United States of America

**James Morgan**

United Kingdom

**Dave Morgan**

United States of America

**Kouichi Morita**

Japan

**Melissa Moss**

United States of America

**Bruno Mourvillier**

France

**Elliott Mufson**

United States of America

**Angelika Mühlebner**

Netherlands

**Rob Mullins**

United States of America

**Benjamin Murdock**

United States of America

**Erland Nagelhus**

Norway

**Istvan Nagy**

United Kingdom

**Matthias Nahrendorf**

United States of America

**Ranuka Nair**

India

**Kunihiro Nakai**

Japan

**Hideaki Nakajima**

Japan

**Mike Namaka**

Canada

**Roland Nau**

Germany

**Christian Naus**

Canada

**Lucia Negri**

Italy

**Andrew Neilson**

United States of America

**Vivek Nerurkar**

United States of America

**Eric Ng**

United States of America

**Michael Nichols**

United States of America

**Robert Nickells**

United States of America

**Robert Nitsch**

Germany

**Christopher Norris**

United States of America

**Kelly Nudelman**

United States of America

**M. Kerry O'banion**

United States of America

**Birgit Obermeier**

United States of America

**Jason O'connor**

United States of America

**Janis O'donnell**

United States of America

**Halina Offner**

United States of America

**Toru Ogata**

Japan

**U Oh**

United States of America

**Andreas Ohlmann**

Germany

**K Ohno**

Japan

**Toshio Ohshima**

Japan

**Johanna Ojala**

Finland

**Tatsusada Okuno**

Japan

**John Olschowka**

United States of America

**Julie Olson**

United States of America

**Benjamin Ondruschka**

Germany

**Hiroaki Ooboshi**

Japan

**Lisa Opanashuk**

United States of America

**Gregory Ordway**

United States of America

**Michael Osthoff**

Switzerland

**Trevor Owens**

Denmark

**Yoko Ozawa**

Japan

**Rosana Pagano**

Brazil

**Guylene Page**

France

**Kalipada Pahan**

United States of America

**Maria Panaro**

Italy

**Alberto Panerai**

Italy

**Francesco Parmeggiani**

Italy

**Rimma Parnova**

Russian Federation

**Guilio M. Pasinetti**

United States of America

**Cesare Patrone**

Sweden

**Gesine Paul**

Sweden

**Prasit Pavasant**

Thailand

**Krystyna Pawlak**

Poland

**Marcela Pekna**

Sweden

**Rosalia Pellitteri**

Italy

**Luis Penalva**

United States of America

**Yu-Ping Peng**

China

**Keith Pennypacker**

United States of America

**Liyanage Perera**

United States of America

**Karin Peterson**

United States of America

**A Petit-Paitel**

France

**Ashley Petrone**

United States of America

**Mary Petzke**

United States of America

**Mario Philipp**

United States of America

**Joanna Phillips**

United States of America

**Irene Pichler**

Italy

**Christian Pike**

United States of America

**Kelly Pike**

Canada

**Sudheesh Pilakka Kanthikeel**

United States of America

**Paulo Pires**

United States of America

**Anna Planas**

Spain

**Patrizia Pontisso**

Italy

**Dean Pountney**

Australia

**David Powell**

United States of America

**Elizabeth Powell**

United States of America

**Christopher Power**

Canada

**Domenico Pratico**

United States of America

**Darwin Prockop**

United States of America

**Barbara Przewlocka**

Poland

**Chaim Putterman**

United States of America

**Zenaide Quezado**

United States of America

**Richard Quigg**

United States of America

**Andre Quincozes-Santos**

Brazil

**P Rakoczy**

Australia

**Glenn Rall**

United States of America

**M. Ramanathan**

India

**Matt Ramer**

United States of America

**Ana Ramirez**

Spain

**G. Rebeck**

United States of America

**Raymond Regan**

United States of America

**William Regenold**

United States of America

**Theo Rein**

Germany

**Markus Reindl**

Austria

**Tonia Rex**

United States of America

**Susanna Ricci**

Italy

**Gianfranco Risuleo**

Italy

**Fatima Rivera**

United States of America

**Phillip Rivera**

United States of America

**Robert Roach**

United States of America

**Mayela Rodriguez**

Mexico

**Moses Rodriguez**

United States of America

**Mary-Louise Rogers**

Australia

**Gu Seob Roh**

Republic of Korea

**Troy Rohn**

United States of America

**Julian Romero**

Spain

**Lorenzo Romero-Ramirez**

Spain

**M Romero-Ramos**

Denmark

**Christopher Rose**

Canada

**Anna Rosell**

Spain

**Thad Rosenberger**

United States of America

**Susanna Rosi**

United States of America

**Kharah Ross**

United States of America

**Joachim Roth**

Germany

**Steven Roth**

United States of America

**Guido Rubboli**

Denmark

**Marc Ruitenberg**

Australia

**Christoph Rummel**

Germany

**Klemens Ruprecht**

Germany

**Horea Rus**

United States of America

**Karsten Ruscher**

Sweden

**Jong Ryu**

Republic of Korea

**Daniel Saban**

United States of America

**Roger Sabbadini**

United States of America

**Ahmad Salehi**

United States of America

**Samina Salim**

United States of America

**Paul Salvaterra**

United States of America

**Tarek Samad**

United States of America

**Vanesa Sanchez-Guajardo**

Denmark

**Juergen Sandkuehler**

Austria

**Lauren Sansing**

United States of America

**Abel Santamaria**

Mexico

**Ana Santiago**

Portugal

**Michael Santillo**

United States of America

**Thomas Santoro**

United States of America

**Rebecca Sappington**

United States of America

**Manuel Sarasa**

Spain

**Sohobhan Sarkar**

Canada

**Harvey Sarnat**

Canada

**Norman Saunders**

Australia

**Josep Saura**

Spain

**Valentina Savchenko**

United States of America

**Cristoforo Scavone**

Brazil

**Eliana Scemes**

United States of America

**Tanja Schlereth**

Germany

**Jürgen Schneider- Schaulies**

Germany

**Herbert Schwarz**

Singapore

**Guillaume Sebire**

Canada

**Jose Segovia**

Mexico

**Fatima Sehba**

United States of America

**Miho Sekiguchi**

Japan

**Dana Selley**

United States of America

**Bridgette Semple**

Australia

**Mrudang Shah**

United States of America

**Zahoor A. Shah**

United States of America

**Lee Shapiro**

United States of America

**Hari Sharma**

Sweden

**Hayley Shawn**

Canada

**Kazim Sheikh**

United States of America

**Jiangang Shen**

China

**John Sheridan**

United States of America

**T Shin**

Republic of Korea

**Seiji Shioda**

Japan

**Esther Shohami**

Israel

**Veronica Shubayev**

United States of America

**Rafael Simo**

Spain

**Roger Simon**

United States of America

**Dario Siniscalco**

Italy

**Luigi Sironi**

Italy

**Stephen Skaper**

Italy

**Thomas Skripuletz**

Germany

**Ann Smith**

United States of America

**Jessica Snowden**

United States of America

**Tomas Sobrino**

Spain

**Betty Soliven**

United States of America

**Volkan Solmaz**

Turkey

**Claudia Sommer**

Germany

**Yoshifumi Sonobe**

Japan

**Linda Sorkin**

United States of America

**Lydia Sorokin**

Germany

**Rosa Sorrentino**

Italy

**Lirlandia Sousa**

Brazil

**Peter Spaeth**

Switzerland

**James St John**

Australia

**Philip Stahel**

United States of America

**Martin Stangel**

Germany

**Larry Steinman**

United States of America

**Mark Stettner**

Germany

**Jonathan Stiles**

United States of America

**Jonathan Stone**

Australia

**T Stone**

United Kingdom

**Barbara Stonestreet**

United States of America

**Wolfgang J. Streit**

United States of America

**Tatyana Strekalova**

Netherlands

**Stephen Strom**

Sweden

**Hyeon-Sook Suh**

United States of America

**Hyeon-Sook Suh**

United States of America

**Kyoungho Suk**

Republic of Korea

**Grace Sun**

United States of America

**Stevens Susan**

United States of America

**Tobias Suter**

Switzerland

**Raymond Swanson**

United States of America

**Fabian Szepanowski**

Germany

**Patricia Szot**

United States of America

**Björn Tackenberg**

Germany

**Kanda Takashi**

Japan

**Shao-Jun Tang**

United States of America

**Malu Tansey**

United States of America

**Maki Tateyama**

Japan

**Juliet Taylor**

Australia

**Elena Tchougounova**

Sweden

**Irmgard Tegeder**

Germany

**Peter Teismann**

United Kingdom

**Andrea Tenner**

United States of America

**Niccolo Terrando**

United States of America

**Charlotte Teunissen**

Netherlands

**Dietmar Thal**

Germany

**Praveen Thumbikat**

United States of America

**Leonardo Tonelli**

United States of America

**Lars Tonges**

Germany

**Terrence Town**

United States of America

**Corinna Trebst**

Germany

**Marie-Eve Tremblay**

Canada

**James Tsai**

United States of America

**Kuen-Jer Tsai**

Taiwan Republic of China

**Styliani Tsirka**

United States of America

**Alexander Tsiskaridze**

Georgia

**Erdem Tuzun**

Turkey

**Neetu Tyagi**

United States of America

**Suresh Tyagi**

United States of America

**Kenneth Tyler**

United States of America

**William Tyor**

United States of America

**Marta Valcarcel-Ares**

United States of America

**Luc Vallieres**

Canada

**Bart Van Berckel**

Netherlands

**Anne-Marie Van Dam**

Netherlands

**Linda Van Eldik**

United States of America

**Barbara Van Leeuwen**

Netherlands

**E. Van Vliet**

Netherlands

**Pamela Vandevord**

United States of America

**Evros Vassiliou**

United States of America

**Elisabetta Vegeto**

Italy

**Claudia Verderio**

Italy

**Annamaria Vezzani**

Italy

**Carl-Wilhelm Voge;**

United States of America

**Zvi Vogel**

Israel

**Christina Vogelaar**

Germany

**Cinzia Volonte**

Italy

**Bruce Volpe**

United States of America

**Joseph Volpe**

United States of America

**Hans-Christian Von Budingen**

United States of America

**Lucy Vulchanova**

United States of America

**Sheela Vyas**

France

**Christian Waeber**

Ireland

**David Wagner**

United States of America

**Amy Wagner**

United States of America

**Adam Walker**

Australia

**Rohan Walker**

Australia

**Zhe Wang**

China

**Guanghu Wang**

United States of America

**Xiaoyang Wang**

Sweden

**X Wang**

China

**Clemens Warnke**

Germany

**James Washek**

United States of America

**John Watt**

United States of America

**Jyoti Watters**

United States of America

**Christine Webber**

Canada

**Sven Wehner**

Germany

**Jonathan Weinstein**

United States of America

**Martha Welch**

United States of America

**Han-Rong Weng**

United States of America

**Gary Wenk**

United States of America

**Andrew West**

United States of America

**Fletcher White**

United States of America

**Scott Whittemore**

United States of America

**Marlene Wiart**

France

**Heinz Wiendl**

Germany

**Donna M Wilcock**

United States of America

**Brigitte Wildemann**

Germany

**James Wiley**

Australia

**Jennifer Wilkinson-Berka**

Australia

**Kenneth Williams**

United States of America

**Didier Wion**

France

**Thomas Wisniewski**

United States of America

**Martin Witt**

Germany

**Yl Wu**

Taiwan Republic of China

**Heping Xu**

United Kingdom

**Fu-Shan Xue**

China

**Koji Yamanaka**

Japan

**Chul-Su Yang**

Republic of Korea

**Shu Yang**

United States of America

**Qing-Wu Yang**

China

**Honghong Yao**

China

**Elke Ydens**

Belgium

**Midori Yenari**

United States of America

**Gelareh Zadeh**

Canada

**Nahla Zaghloul**

United States of America

**Guang-Xian Zhang**

United States of America

**Ji Zhang**

Canada

**Jun-Ming Zhang**

United States of America

**Yumin Zhang**

United States of America

**Zheng Zhang**

United States of America

**Xuechu Zhen**

China

**Wendy Ziai**

United States of America

**Gregory Zipfel**

United States of America

**Berislav Zlokovic**

United States of America

**Robert Zorec**

Slovenia

**Zhiyi Zuo**

United States of America

